# A satellite imagery-driven framework for rapid resource allocation in flood scenarios to enhance loss and damage fund effectiveness

**DOI:** 10.1038/s41598-024-69977-1

**Published:** 2024-08-20

**Authors:** Jeremy Eudaric, Heidi Kreibich, Andrés Camero, Kasra Rafiezadeh Shahi, Sandro Martinis, Xiao Xiang Zhu

**Affiliations:** 1 Chair of Data Science in Earth Observation, Department of Aerospace and Geodesy, Technical University of Munich, 80333 Munich, Germany; 2 Earth Observation Center, German Aerospace Center (DLR), 82234 Wessling, Germany; 3 Section Hydrology, GFZ German Research Centre for Geosciences, 14467 Potsdam, Germany; 4 Munich Center for Machine Learning, 80333 Munich, Germany

**Keywords:** Natural hazards, Hydrology, Computer science

## Abstract

The impact of climate change and urbanization has increased the risk of flooding. During the UN Climate Change Conference 28 (COP 28), an agreement was reached to establish “The Loss and Damage Fund” to assist low-income countries impacted by climate change. However, allocating the resources required for post-flood reconstruction and reimbursement is challenging due to the limited availability of data and the absence of a comprehensive tool. Here, we propose a novel resource allocation framework based on remote sensing and geospatial data near the flood peak, such as buildings and population. The quantification of resource distribution utilizes an exposure index for each municipality, which interacts with various drivers, including flood hazard drivers, buildings exposure, and population exposure. The proposed framework asses the flood extension using pre- and post-flood Sentinel-1 Synthetic Aperture Radar (SAR) data. To demonstrate the effectiveness of this framework, an analysis was conducted on the flood that occurred in the Thessaly region of Greece in September 2023. The study revealed that the municipality of Palamas has the highest need for resource allocation, with an exposure index rating of 5/8. Any government can use this framework for rapid decision-making and to expedite post-flood recovery.

## Introduction

Climate change has increased the frequency and severity of flood hazards worldwide^1^. Recent studies highlight the importance of local spatial development choices in determining community exposure to flood hazards^2^. Satellite imagery reveals a projected increase in the proportion of the population exposed to flood events by 2030^3^. The economic consequences of flood risks are substantial, with an estimated 9.8 trillion US dollars of economic activity directly located in areas with significant flood risks^4^. Moreover, at a global warming scenario of 2 ${}^{\circ }$C, the projected direct economic losses are expected to double^5^. In response to these escalating economic losses resulting from climate change, COP 28 introduced a significant advancement by establishing “The Loss and Damage Fund” for low-income and vulnerable countries^6^. However, allocating funding can often be slow and inefficient^7^. Limited data availability and unclear frameworks for distributing resources pose challenges^7,8^. In the context of worldwide climate change, it is capital to rapidly determine which regions require funds and when and where these funds should be allocated after a flood event^7,9^. This assessment is necessary to facilitate effective public policy decisions, allocate budgets, and distribute resources for compensation purposes^9–11^.

Evaluating resources for emergency response can only occur after natural hazard events when damage and the population affected can be recorded^12^. Rapidly monitoring flood hazards is essential for a more accurate assessment close to the peak discharge^12,13^. Satellite images are a valuable source of information for monitoring various risks, including flood hazards and exposure^3^. Most floods are monitored through international disaster response mechanisms such as the Copernicus Emergency Management Service (CEMS) with satellite systems. However, CEMS does not provide a clear resource allocation framework for emergency reconstruction and refunding after a flood event. In our study, we used Sentinel-1 and Sentinel-2 for flood mapping because of their availability to the public at no cost, the duration over which they provide data, and their established effectiveness in monitoring water and flood dynamics. Both Synthetic Aperture Radar (SAR) and optical images can assist in mapping flood events. With optical images from Sentinel-2, only about one-fifth of the event can be observed, whereas with Sentinel-1, approximately three-fifths can be captured^14^. During nighttime, the absence of natural light sources, such as sunlight, can make it challenging for optical sensors to capture clear and detailed images of flooded areas. The limited availability of light can result in darker and less distinguishable images.

A Deep Learning (DL) framework has been developed to propose a comprehensive assessment based on flood mapping using optical satellite imagery^15^. This framework involves per-pixel segmentation, considering the probability of cloud cover and the probability of water presence. Yet, challenges arise when using optical images due to the unpredictable presence of clouds near the flood peak. The accuracy of cloud removal techniques depends on cloud thickness and semi-transparency, which could lead to a less accurate assessment^16^. Using DL for SAR images is possible but challenging due to the spectral noise of interference^17,18^. Labeling data for a model in emergency scenarios could pose challenges due to the scarcity of labeled data and the cost, time-consuming nature, and error-prone process of data annotation, which hinders appropriate training^18,19^.

**Figure 1:**
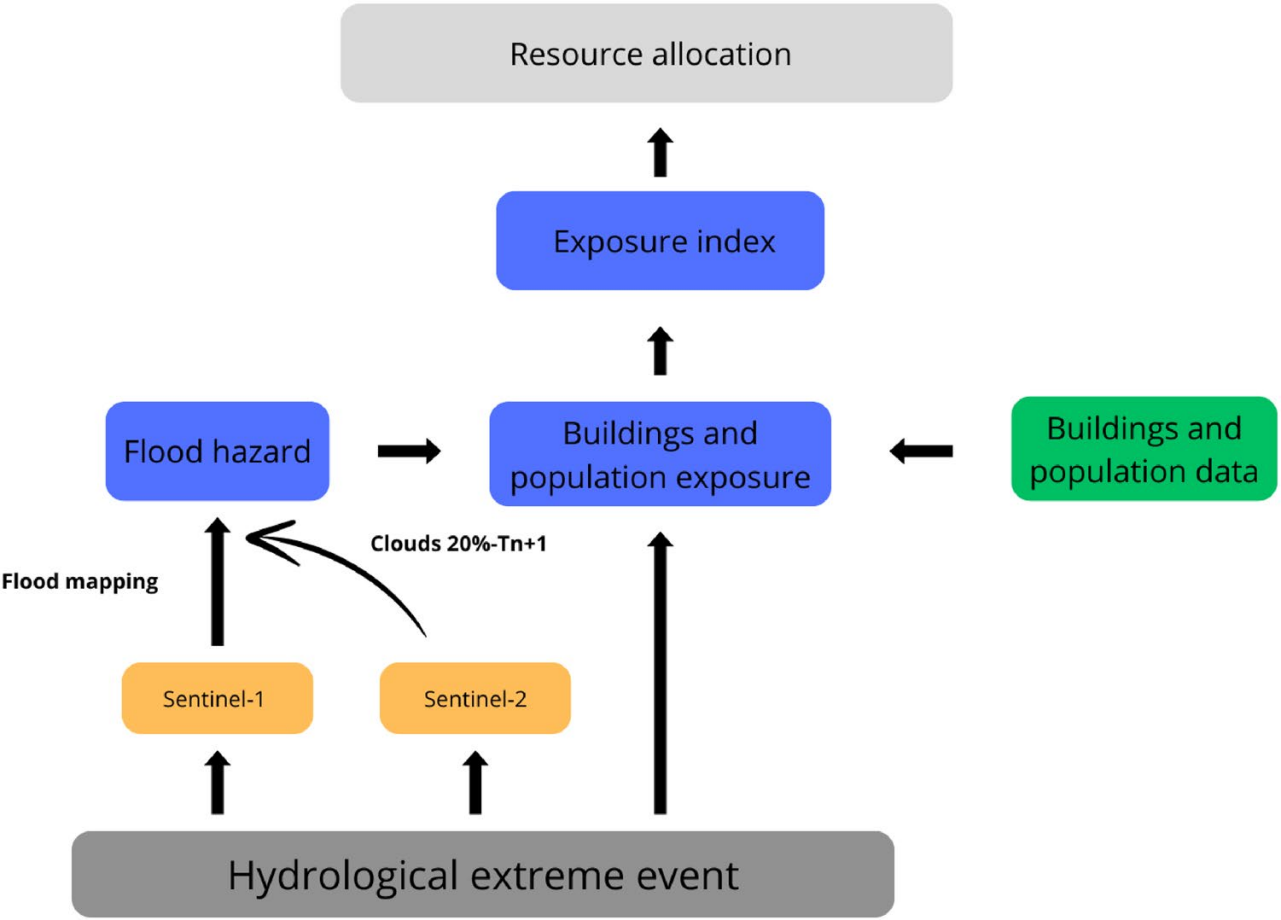
Framework rapid resource allocation in flood scenarios. The figure represents the global method based on flood mapping with Sentinel-1 (SAR images) and Sentinel-2 (optical images) for validation. Thus, the EI is created based on satellite images of flood hazards, buildings, and population exposure.

To address the challenges in this study, we propose a novel resource allocation framework. In contrast to a previous study that relied solely on population density^20^, our research introduces an exposure index (EI) based on satellite imagery and geospatial data (see Fig.1). Our framework can be applied to analyze flood events lasting only several days using free satellite images. Sentinel-1 SAR images are used to map floods near the peak. The Sentinel-1 mission’s microwave signal can penetrate through clouds and operate during nighttime^14^. Sentinel-2 images are collected under better cloudy conditions to validate and evaluate the inundation. The EI allows a comprehensive approach, encompassing economic (buildings) and social (population) aspects (see Fig. 1), combining flood hazard, building exposure, and population exposure. We assess flood hazard by utilizing the flood map. Buildings and population exposure are evaluated based on flood hazards using buildings and population density data. The EI enables the government to take a holistic view and determine how resources should be allocated based on exposure severity. In September 2023, Greece experienced a devastating flood caused by heavy rainfall in the frame of Storm Daniel. We used this event as a case study to test our method. The following sections will delve into the methodology and results of our work.

**Figure 2:**
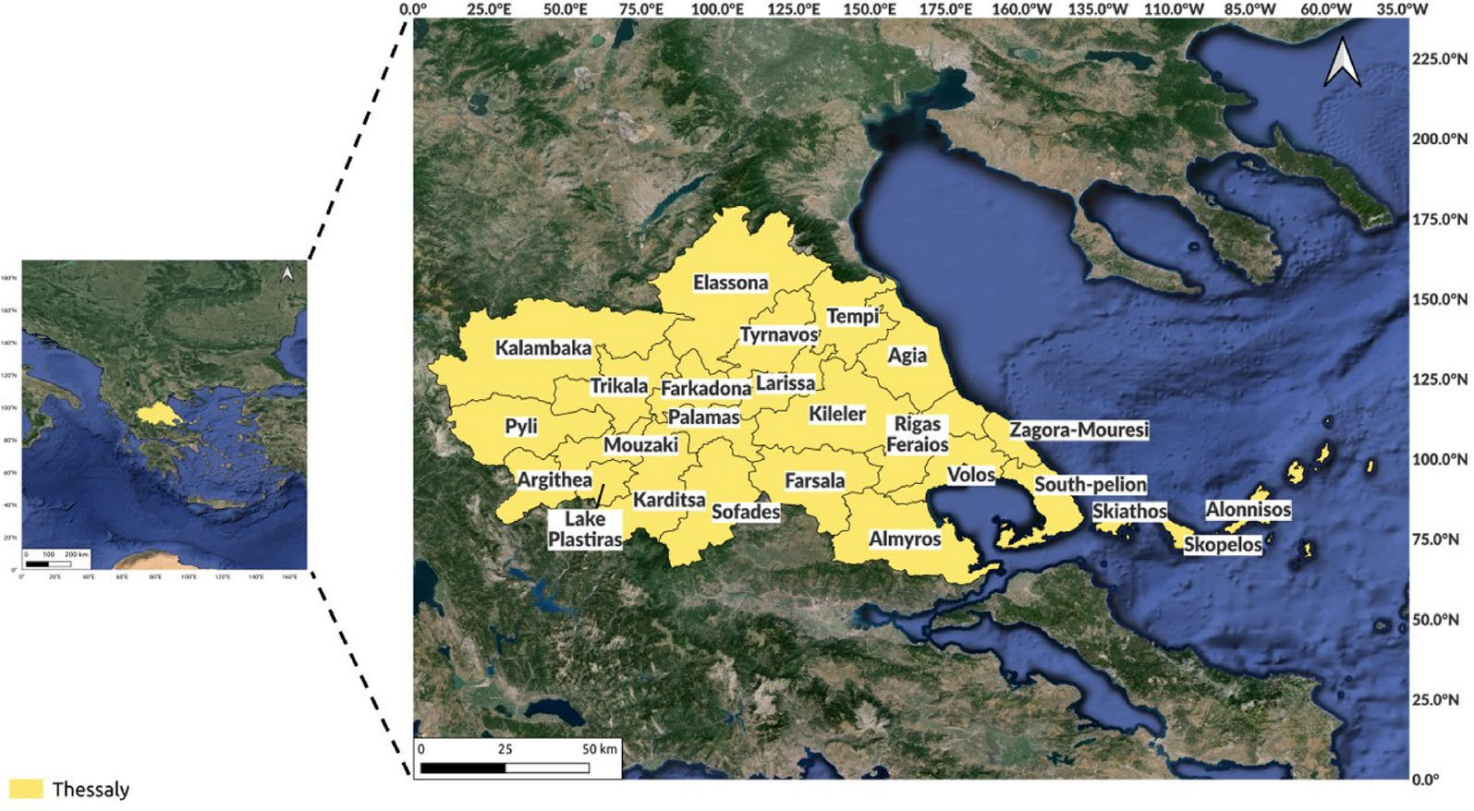
Map of the Thessaly region, produced using quantum GIS (QGIS)^21^.

## Study area and data set

### Study area

Greece exhibits one of the lowest GDPs in the European Union. The region of Thessaly in Greece comprises 25 municipalities and covers an area of 1,403,600 hectares (see Fig. 2). On September 4 2023, a severe flood occurred due to heavy rainfall, marking the most extreme rainfall event in Greece’s recorded history. The damages sustained in the Thessaly region alone were estimated to range between 1.5 billion and 2.5 billion euros. The Thessalian plains in Greece are the primary agricultural hub, contributing approximately 12.2% to the country’s farming industry’s gross value added^22^.

### Data description

#### Satellite data

Natural hazard mapping can be conducted using satellite images. We used two satellite missions, Sentinel-1 for flood mapping and Sentinel-2 as a reference for validation purposes. These two satellites were launched by the European Space Agency (ESA). The selection of satellites is based on their public availability, which promotes reproducibility, transparency, and accessibility in research. This ensures that governments and insurance agencies can utilize the model without limitations. We collected all Sentinel data via the Copernicus Data Space Ecosystem^23^.

Sentinel-1 SAR data: Sentinel-1A was launched on April 3, 2014, and Sentinel-1B was launched on April 25, 2016. This satellite constellation provides SAR imagery of Earth day or night, regardless of weather conditions, with a revisit time of 6 days at the equator^24^. Sentinel-1B is out of service, resulting in a revisit time of only 12 days. This study uses Sentinel-1 Interferometric Wide Swath (IW) dual polarized (VV, VH) data with a 250 km swath at a spatial resolution of 5 m by 20 m in single look complex (SLC) format.

Sentinel-2 optical data: Sentinel-2A was launched on June 23, 2015, and Sentinel-2B was launched on March 7, 2017. The MultiSpectral Instrument (MSI) on the Sentinel-2 satellite captures data in 13 spectral bands, with four bands at a spatial resolution of 10 m, six bands at 20 m, and three at 60 m. The satellite has a revisit time of 5 days at the equator^25^. The study used the Level-2A product, which provides atmospherically corrected Surface Reflectance (SR) images.

#### Administrative boundaries

The scale is an essential factor for allocating resources. We have chosen the municipality scale and, therefore, utilized the OpenStreetMap (OSM)^26^ dataset and DIVA-GIS^27^ to obtain the administrative boundaries of each municipality.

#### Population data

We estimated the population density with the WorldPop High-Resolution Population dataset (WPGP), created by the University of Southampton^28^. This dataset provides detailed and open-access spatial demographic information, including the number of inhabitants per cell, with a resolution of 3 arcseconds. The global coverage of this dataset spans from 2000 to 2020. The population estimations are available at approximately 100 m and 1 km resolution for 2020, along with estimates of the number of people belonging to individual age-sex groups. In our study, we have chosen a resolution of 100 m grid population.

#### Buildings data

We utilized the Microsoft Buildings Footprints^29^ dataset to estimate the number of buildings. This dataset provides a digital representation of building outlines derived from high-resolution optical Maxar satellite images and processed using deep learning algorithms. In total, 1.3 billion buildings were detected between 2014 and 2023.

## Results

### Flooded area

**Figure 3:**
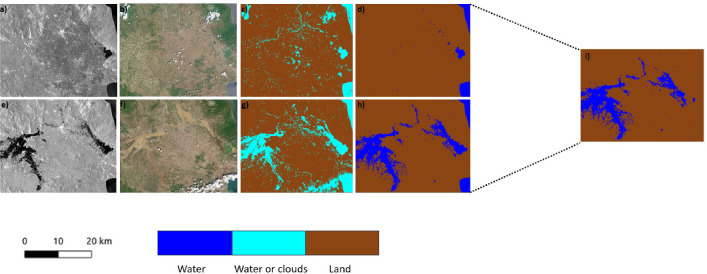
Flood mapping in Thessaly region using satellite imagery (a big part of the image). **(a)** Pre-disaster Sentinel-1 image taken on June 27, 2023. (**b)** Pre-disaster Sentinel-2 image taken on June 22, 2023. **(c)** Pre-disaster Sentinel-2 validation data segmented based on the NDWI. **(d)** Pre-disaster Sentinel-1 data is classified with the standard deviation threshold. **(e)** Post-disaster Sentinel-1 image taken on September 7, 2023, represents the flood peak. **(f)** Post-disaster Sentinel-2 image taken on September 10, 2022. **(g)** Reference co-disaster Sentinel-2 data segmented based on the NDWI. **(h)** Co-disaster Sentinel-1 with the standard deviation threshold. **(i)** Change detection represents the result of pre and post-disaster images. The scale bar has been produced using quantum GIS (QGIS)^21^.

We used the post-disaster image with the most water pixels to assess the flood’s peak (see “Methodology”). Sentinel-1 images were collected before the disaster on June 27, 2023, and after the disaster on September 7, 2023, with an ascending orbit. Additionally, Sentinel-2 images were collected before the disaster on June 22, 2023, and after the disaster on September 10, 2023 (Fig. 3). Challenges arise when attempting to collect all flooded area data at once from Sentinel-1 due to the vast extent of the flood. Hence, we divided the images into two parts: the first part is the central part, and the second is a tiny portion of the flood extension. We generated a flooded map using the SAR images by applying the four thresholding techniques mentioned in the Methodology on both parts of the image. To evaluate the performance, we used Band B3 (green) and B8 (near-infrared) from Sentinel-2 with cloud coverage $\le 20$ % to get the Normalized Difference Water Index (NDWI) used for validation (see “Methodology”). The optical images were divided into two parts and collected in better weather conditions. When applying the NDWI, we observed difficulties in making a clear distinction between clouds and water as presented in Fig. 3. Clouds and water bodies often share similar spectral characteristics, exhibiting high reflectance in the visible spectrum. This similarity can make clouds appear bright on satellite optical imagery. Our analysis highlights Standard Deviation as the best approach for segmenting water and creating the best water pixel mask (on the first part of the image and the tiny part only of the pre-disaster image). We can see the overall results in Tables 1 and 2. The Standard Deviation is a suitable threshold for this analysis due to the observed normal distribution of the Digital Number (DN), as shown in the “Methodology” section. In a Gaussian distribution, most of the grey level values for water pixels are clustered around a central value, with fewer values deviating significantly from the mean. By considering the Standard Deviation, we can assess the spread of the data and distinguish between water and non-water. The Triangle method effectively segmented the post-disaster area of the smaller portion of the image, as highlighted in Table 2. This result is potentially due to the DN distribution of the image, which does not have an apparent Gaussian curve. Hence, we can use the change detection method explained in “Methodology” to get a flooded map for each image part (Fig. 3). We have assembled both flood maps and applied a threshold to obtain the flooded map. We then used this flooded map to create the final flooded mask (Fig. 3).

**Table 1:** Results of the intersection over union (IoU) and recall (see “Methodology”) for the images before and after the disaster, specifically for the larger portion of the image.

	IoU pre-disaster	IoU post-disaster	Recall pre-disaster	Recall post-disaster
Otsu	0.44	0.89	0.70	0.94
Triangle	0.91	0.90	0.95	0.94
Standard deviation	**0.96**	**0.91**	**0.97**	**0.95**
Threshold minimum	0.67	0.60	0.85	0.84

Significant values are in bold.

**Table 2:** Results of the intersection over union (IoU) and recall (see “Methodology”) for the images before and after the disaster, specifically for the smaller portion of the image.

	IoU pre-disaster	IoU post-disaster	Recall pre-disaster	Recall post-disaster
Otsu	0.38	0.70	0.65	0.85
Triangle	0.83	**0.81**	0.92	**0.91**
Standard deviation	**0.95**	0.75	**0.96**	0.89
Threshold minimum	0.53	0.65	0.77	0.84

Significant values are in bold.

**Figure 4:**
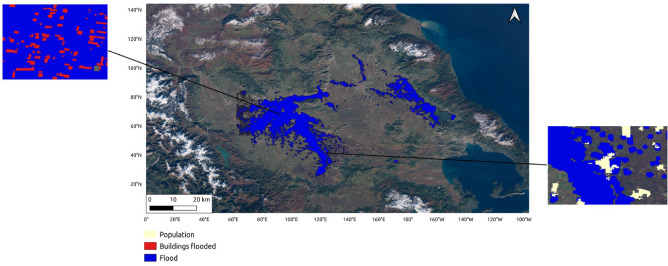
The final flooded map is based on the assembly of the two parts of the SAR image. Example of buildings exposure based on the flood map and buildings data in the municipality of Palamas. Population exposure based on the flood mask and population data in Farsala municipality. The map has been produced using Quantum GIS (QGIS)^21^.

### Results

#### Flood hazard

**Figure 5:**
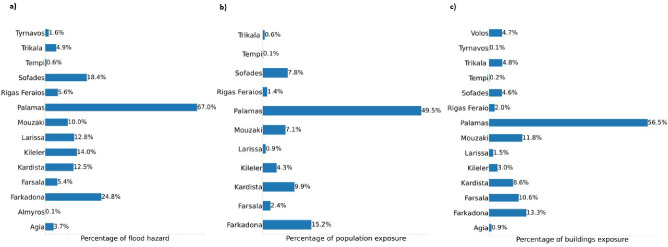
**(a)** Flood hazard. **(b)** Population exposure. **(c)** Buildings exposure.

**Figure 6:**
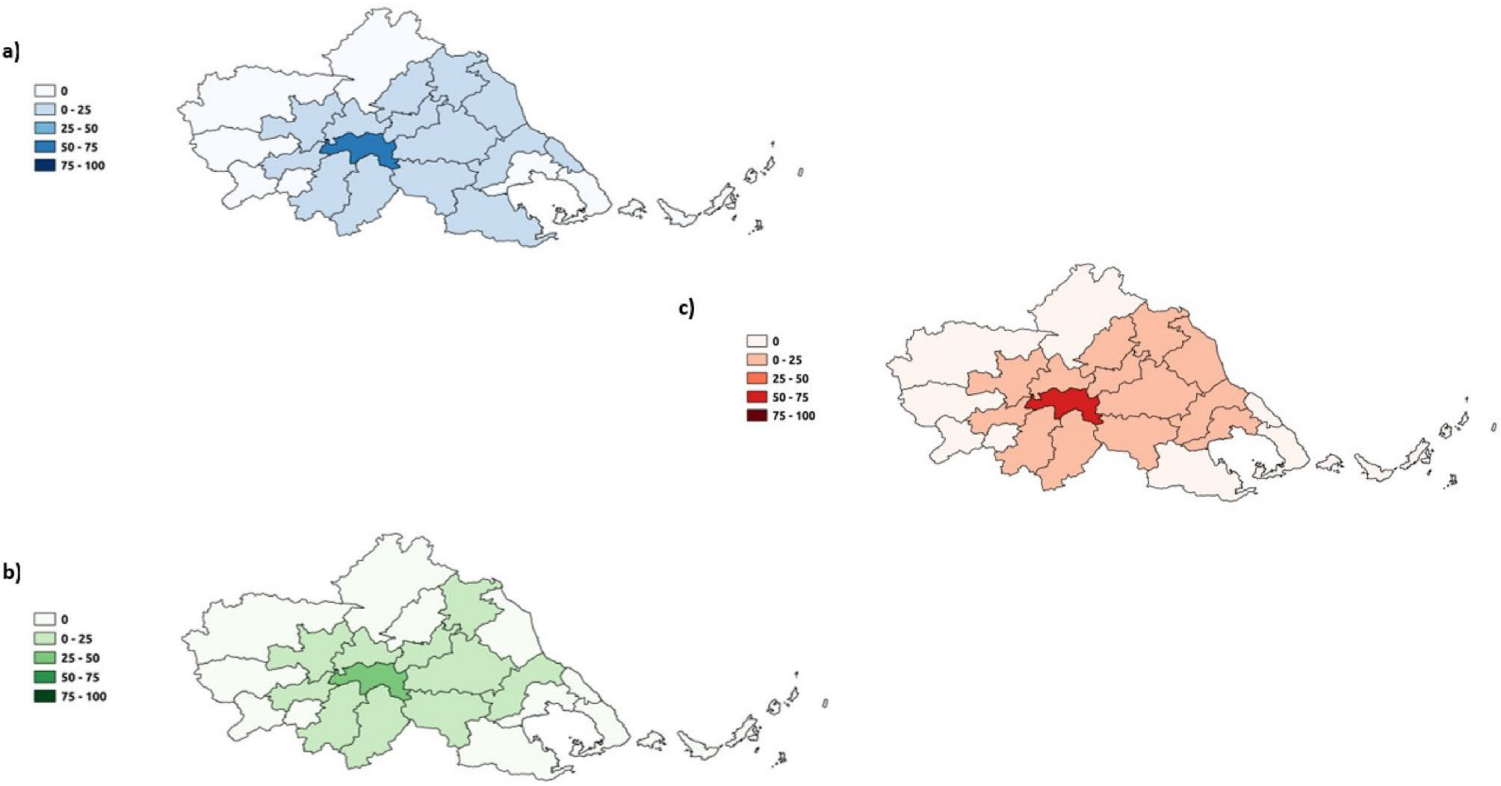
Results are categorized into five distinct severity percentage intervals: “Not affected”, “Minor affected”, “Affected”, “Major affected”, and “High affected”. **(a)** Flood hazard. **(b)** Population exposure. **(c)** Buildings exposure. These maps have been produced using quantum GIS (QGIS)^21^.

We conducted a municipality-level flood hazard assessment to evaluate the extent and severity of flooding in the area. Administrative boundaries were obtained from OSM and DIVA-GIS. We then overlaid the flooded pixels map with the administrative boundaries to determine the local flooded area and calculated the percentage of flood hazard for each municipality, as shown in Fig. 4. Our analysis revealed that the municipalities of Palamas and Farkadona were the most affected by the flood hazard, with 67.0% and 24.8% (Fig. 5) of their areas, respectively, experiencing flooding near the peak of the flood. In the region, we found an average flood hazard of 7.7%. Based on these findings, the flood hazard for the municipality of Palamas was categorized as “Major affected” (Fig. 6). In contrast, the municipality of Farkadona was categorized as “Minor affected” (Fig. 6). These results highlight Palamas and Farkadona as the municipalities where the flood hazard is higher, potentially, urban centers, population, buildings, and other land uses.

#### Buildings exposure

The estimation of building exposure utilizes the Microsoft Building Footprints dataset^29^, which offers a comprehensive collection of worldwide building footprints. We identified the flooded buildings by overlaying the pixels map with the building footprints (Fig. 4). Notably, near the peak discharge of the flood, the municipality of Palamas emerged as the most affected, with 56.5% of buildings exposed, followed by Farkadona at 13.3% (Fig. 5). In the Palamas region, a correlation was observed between flood hazard and building exposure, both categorized as “Major affected”. Additionally, the municipality of Sofades exhibited an 18.4% higher flood hazard compared to a 4.6% building exposure, both classified as “Minor affected” Figs. 5 and 6. Upon deeper analysis, it became apparent that the municipality of Mouzaki demonstrated similarity in the “Minor Affected” category for both flood hazard and building exposure. However, the percentage of building exposure, at 11.8% (Fig. 5), exceeded the flood hazard percentage of 10% (Fig. 5). This result could indicate that while flood hazard and building exposure categories may align, the severity percentages can vary. The average building exposure in the region is 4.9%.

#### Population exposure

**Figure 7:**
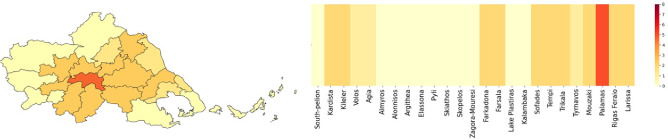
Exposure index. Resource allocation for each municipality. The Thessaly map has been produced using quantum GIS (QGIS)^21^.

We conducted a population exposure analysis using the WPGP dataset, which provides data with a resolution of 3 arcseconds, equivalent to approximately 100 m at the equator. WPGP allowed us to improve the granularity of our analysis^4^. Our study focused on Greece and used the most recent population estimates from the United Nations for 2020. By overlaying the population map with the flooded pixel map (see Fig. 4), we calculated the severity percentage of the population affected using a zonal statistic approach^21^. Our research findings indicate Palamas and Farkadona municipalities exhibit the highest population exposure levels. The percentage of population exposure in Palamas was 49.5%, while 15.2% in Farkadona (see Fig. 5). In Palamas, the population exposure is categorized as “Affected” (see Fig. 6). In Farkadona, the flood hazard was categorized as “Minor affected” with a percentage of 24.8%, white the population exposure as categorized as “Minor Affected” with a percentage of 15% (see Figs. 5 and 6). In the municipality of Trikala, the flood hazard is higher than the population exposure percentages, which were 4.9% and 0.6% respectively, both categorized as “Minor Affected” (see Figs. 5 and 6). A similar pattern was observed in the municipality of Larissa, where the flood hazard and population exposure percentages were 12.8% and 0.9% respectively, both categorized as “Minor affected” as observed in Figs. 5 and 7. In Kardista, there was a correlation between the flood hazard 12.5% and population exposure 9.9%, both categorized as “Minor affected” as observed in Figs. 5 and 6. The overall average population exposure in the Thessaly region is 4.7%. Our analysis indicates an overall correlation between flood hazards and population exposure within the same category. However, a high flood hazard does not necessarily mean that the population will be maximally affected.

#### Exposure index

In this study, we present the results of an EI analysis conducted in the Thessaly region to assess the resources required in the event of a flood. The EI considers the flood hazard and the cumulative impact on buildings and population among the municipalities in the region. Palamas has been identified as having the highest index value of 5/8, as presented in Fig. 7, indicating a significant need for resources following a flood (Fig. 7). The Municipality of Fakadona, Kardista, Kieler, Farsala, Sofades, Tempi, Trikala, Mouzaki, Rigas Feraio, and Larissa have also been identified as requiring rapid minor assistance, with an index value of 2/8 (Fig. 7). The index values were calculated based on surface area, population, and the number of buildings in each municipality. Even if two municipalities have the same index value, the allocation of resources is determined by proportional factors that are specific for each one.

## Discussion and conclusion

In the Thessaly region of Greece, flooding began on September 4, 2023. This study aims to propose a framework that leverages satellite imagery and geospatial data to provide rapid resource allocation in the aftermath of a flood event (at a chosen level). The framework is designed to support climate justice and “The Loss and Damage Fund,” enabling governments to quickly evaluate hydrological events’ economic and social impact, facilitating efficient funding and reconstruction efforts. We tested our method using Greece and the region of Thessaly. To map the flood event, we utilised Sentinel-1 data, which allows for observation close to the peak of the flood regardless of weather conditions, including cloudy scenarios. Our flood mapping methods, compared to CEMS Rapid Mapping, assess the flood area simply, accurately, and quickly. This is crucial in an emergency response context. We used Sentinel-2 data as validation to ensure the best flood maps and provide the most accurate resource allocations possible. By creating a flooded area map, we can define the hazard intensity based on the flood hazard and subsequently calculate the EI by incorporating buildings and population data. However, factors such as the presence of similar reflectance properties in other materials (e.g., in urban areas), the type of reflection (specular or diffuse), and the smoothness or texture of the water surface can impact the creation of a binary mask for water and non-water, especially at the city level. The municipality of Palamas stands out with the highest EI, indicating a pressing need for significant resources from the Greek government, especially in emergency scenarios. We observed that a concentration of buildings in specific areas could increase or decrease the exposure. The EI could be influenced by the region’s geographical location and economic activity. Whether the economic diver of a municipality focuses more on the primary economy, such as agriculture, may involve more land flooding than buildings of the population. The flood hazard could be higher than other drivers. This analysis aligns with the geographical and economic status of the region^22^.

The EI can support policy-making at national and international scales by facilitating the rapid allocation of resources. However, insurance coverage rates are typically low, and the EI cannot function independently without a parallel government mechanism^30^. The EI has the potential to enhance collaboration and streamline budget compensation with insurance companies following a disaster. It can also improve disaster management, resource allocation, and resilience to climate-related disasters. For example, during flood events, technical experiments may be rendered inoperable, or in some countries, the quality of fast damage assessment can be limited due to a lack of personnel or infrastructure. In such cases, the availability of free satellite images is crucial. These free data enable countries with limited data access, mainly those less economically advanced, to reproduce the results and ensure equity in decision-making. By leveraging this method, a solidarity fund can be quickly distributed to impoverished countries, facilitating swift humanitarian responses to climate-related disasters like floods.

In conducting efficient assessments, having accurate and comprehensive building data is crucial. While Microsoft building footprints provide a wide range of information, additional details such as building type (private or non-private) and age are necessary to assess precisely. However, datasets like EUBUCCO for Greece and OSM lack comprehensive building characteristics. For example, only 5% of building heights and 12% of building types are available for the 864,237 buildings in the EUBUCCO dataset^31,32^. The lack of buildings’ characteristics can result in less accurate assessments, particularly in the context of climate change adaptation and mitigation. The absence of comprehensive inventories poses a significant challenge and underscores the importance of developing these inventories, as emphasized by the United Nations Office for Disaster Risk Reduction (UNDRR)^33^. To address this issue, governments should collaborate to improve the completeness and quality of geospatial data. One potential solution is using DL models to make predictions based on high-resolution satellite images. These models can identify buildings, determine their types, and estimate their heights, enhancing the accuracy of building data for assessments.

We found three limitations in this method: (1) Our process is not well-suited for monitoring flash floods due to the mismatch between the time scales of Sentinel-1 and Sentinel-2 revisits and the duration of the floods. The revisit time of Sentinel-1 is 12 days. A significant time gap between observations may result in missing important details or changes in the flood situation during that period. Thus, the flood hazard, buildings, and population exposure could be underestimated close to the flood peak. (2) The lack of building characteristics allows us to estimate only the building’s exposure roughly. (3) Applying the framework at the city level can be challenging due to the difficulty in using Sentinel-1 data in urban areas, primarily because of issues related to reflectance.

For further work, we foresee three directions. Firstly, we could assess vulnerability as crucial to estimate the financial resources required for effective disaster management and model the socio-economic impact. This aspect becomes particularly significant for low-income countries, where limited resources and infrastructure can exacerbate the impacts of natural hazards. By considering vulnerability, resource allocation can be tailored to address specific vulnerabilities and facilitate collaboration between poorer and wealthier nations. Secondly, to enhance flood mapping capabilities, integrating advanced sensors such as TerraSAR-X/TanDEM-X and WorldView-3 can be invaluable. These sensors offer high-resolution imagery and can be particularly useful in mapping flash floods. By incorporating this technology, the accuracy and timeliness of flood mapping can be significantly improved, thereby enabling more effective emergency response strategies. Finally, the inclusion of hydrological data is relevant to improve the framework. Parameters such as water depth, runoff coefficient, water velocity, or meteorological data provide valuable insights into the dynamics of flooding events. Incorporating flood depth data enhances our ability to assess an accurate building exposure, understand how different inundation levels can impact structures, and evaluate flood damages and costs using depth-damage curves^34^. To apply this method, obtaining information about the inundation water depth and building types (residential, commercial, or industrial) is essential. Water depth can be estimated using remote sensing data from inundation maps based on satellite images and a digital elevation model (DEM)^35^. However, the accuracy of these methods can be compromised by challenges such as spatial mismatch between the inundation extent and the DEM and dealing with complex flat topography.

## Methodology

### Flood mapping

#### Inundation map

**Figure 8:**
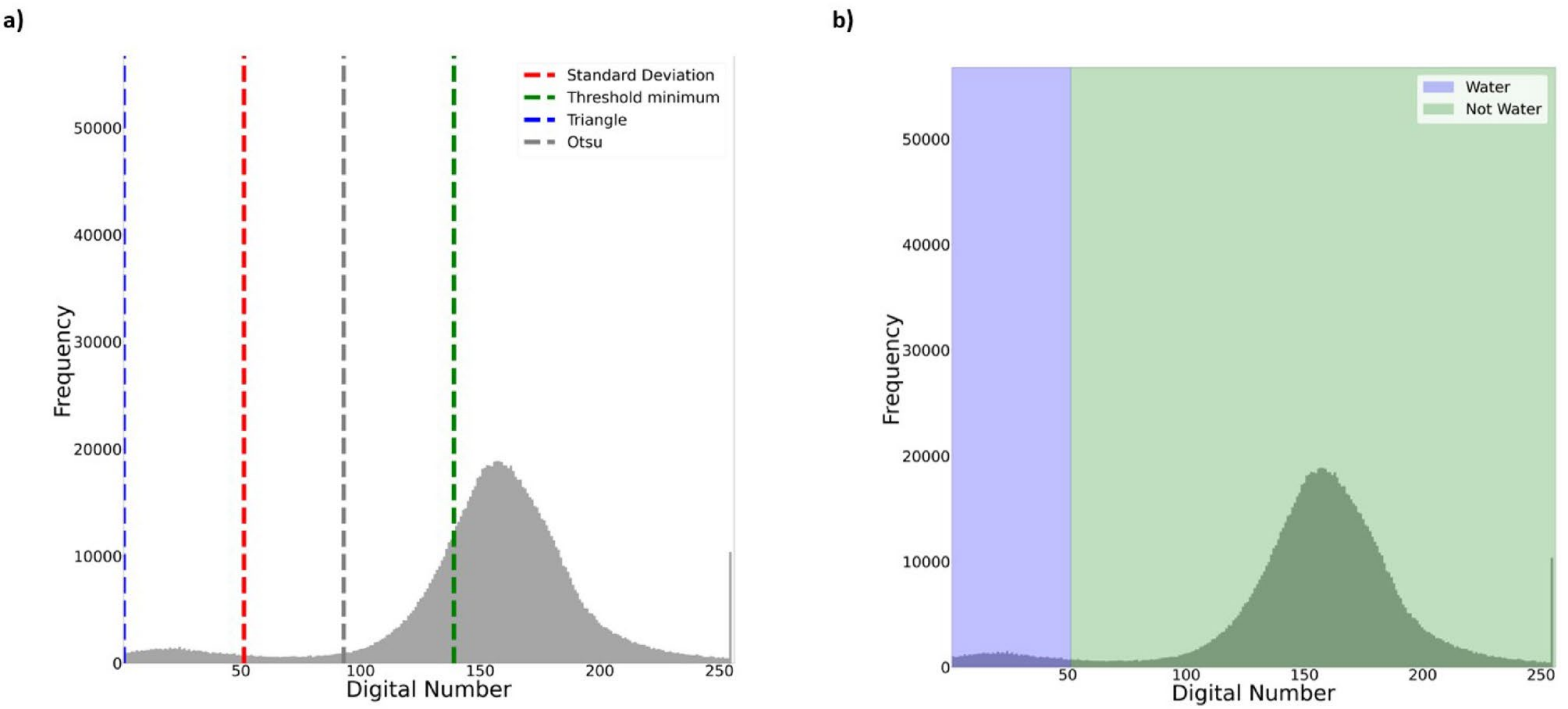
**(a)** Use different thresholds for water segmentation regarding the DN distribution. **(b)** Segmentation water or non-water.

We collected SAR images prepossess from the Copernicus Data Space Ecosystem. Before downloading SAR images, the ecosystem goes through several steps to the images. These steps include thermal noise removal, radiometric calibration, de-bursting, multi-looking, speckle filtering, terrain correction, and orbit file adjustments^36^. The DN represents the intensity of electromagnetic energy measured for the ground resolution cell, represented by each pixel in the image. A high DN for the amplitude of a SAR image pixel represents strong backscatter, while a low DN represents weak backscatter. The specific reflectance characteristics of surface water result in a lower backscatter value, which enables the rapid distinction between the foreground and background in an image and creates a binary mask of water and non-water. Thresholds on SAR images have been used widely in the literature to map the flood hazard^37,38^. Various processing methods can result in variations in the frequency distribution of grey-level values represented as a histogram^39^. The performance of thresholding in SAR image analysis inherently depends on the unique characteristics and properties of the analysed image. Therefore, testing multiple thresholds is crucial for achieving accurate results. In our study, we employ four different thresholding methods to compare images before and after the event: Otsu^40,41^, Triangle^42^, Standard Deviation^43^, and Threshold Minimum^44^ (see Fig. 8). The DN below the threshold is characterised as water and above as non-water (see Fig. 8). We used a pixel-based change detection method to determine the flooded mask. This involved subtracting the pre- and post-disaster images and selecting the Threshold that yielded the best results on each image^45^.

#### Evaluating the inundation map

We can encounter challenges in verifying SAR-based mapping during a flood event in a real scenario due to limited ground truth data and time-consuming annotation for emergency response. In response, we utilized optical images from Sentinel-2 and the NDWI (Eq. 1) to establish an accurate validation. In the window of the flood event, we select the first image with $\le 20$ % clouds-free under better weather conditions. The NDWI is a remote sensing index used to measure used primarily to detect and monitor open water features^46^. This index is calculated using reflectance values from near-infrared (NIR) and Green bands of Sentinel-2.

1 $$\begin{aligned} NDWI = \frac{(Green - NIR)}{{(Green + NIR)}} \end{aligned}$$

We assess the accuracy of the flood map generated using our method by comparing it with the ground truth using two metrics: Intersection over Union (IoU) (Eq. 2) and Recall (Eq. 3). These metrics are defined based on the potential errors and the sensitivity of the binary segmentation mentioned above. True Positive (TP): pixels that are correctly classified as water, False Positive (FP): non-water pixels classified as water, True Negative (TN): pixels that are correctly classified as non-water pixels, False Negative (FN): water pixels classified as non-water.

2 $$\begin{aligned} IoU= & {} \frac{TP}{TP + FP + FN}\end{aligned}$$

3 $$\begin{aligned} Recall= & {} \frac{TP}{TP + FN} \end{aligned}$$

### Hazard and exposure

To effectively resource distributions after a flood event, we need to consider geographical and temporal factors^9^. The variable *i* represents the scale of the study. The variable *t* represents the time of the event closest to the peak of the flood, which captures the maximum impact and allows a comprehensive and efficient assessment. Our goal is to select a post-disaster satellite image near the peak flood discharge to estimate the extent of damage under maximum runoff coefficient conditions^47^. We assumed satellite images with more water pixels correspond to higher peak discharge levels. This assumption is based on the premise that the presence of water pixels indicates a greater volume of water flow. We conducted a comparative analysis of the satellite images obtained in the temporal series of the flood event; we obtained four satellite images in orbit descending and ascending from September 6 until September 13, 2023. For instance, if the number of water pixels in the second image is higher, we use it for the indicators. However, we will apply the assessment only to the second satellite image if the pixel number is lower in the third image.

The hazard refers to the possible event of a natural or human-caused physical occurrence that could lead to loss of life, injury, or health effects^1,20^. The flood hazard ${F}_{\rm it}$, as expressed in the Eq.4 is determined by the extent of flooding at the chosen scale ${F}_{\rm x}$ based on the flooded map and ${F}_{\rm y}$ is the size of the respective area, both measured in hectares (ha).

4 $$\begin{aligned} F_{\rm it} = \frac{F_{\rm x}}{F_{\rm y}} \end{aligned}$$

The exposure refers to the situation of the population or infrastructure in the hazard areas^1,20^. The Buildings exposure $B_{\rm it}$ as described in Eq. (6) is determined by the number of buildings affected by the flood hazard, denoted as $B_{\rm x}$, and the total number of buildings in the area, represented by $B_{\rm y}$.

5 $$\begin{aligned} B_{\rm it} = \frac{B_{\rm x}(F_{\rm x})}{B_{\rm y}} \end{aligned}$$

The population exposure $P_{\rm it}$ (see Eq. 7) is composed of $P_{\rm x}$ the population affected by the flood hazard, and $P_{\rm y}$ represents the total population within the specific scale.

6 $$\begin{aligned} P_{\rm it} = \frac{P_{\rm x}(F_{\rm x})}{P_{\rm y}} \end{aligned}$$

In our analysis, the $F_{\rm it}$, $B_{\rm it}$, and $P_{\rm it}$ results are categorized into five distinct severity percentage intervals, each corresponding to a specific class. The class intervals and their corresponding assigned percentage and class number: equal to 0% (0): “Not affected”, below or equal to 25%, (1): “ Minor Affected”, below or equal to 50%, (2): “Affected”, below or equal to 75%, (3): “Major affected”, below or equal to 100%, (4): “Highly affected”.

The assessment of buildings and population exposure encompasses economic and social aspects. The EI (see Eq. 7) is based on the summation of the assigned number of buildings and population exposure in the chosen scale. Thus, we can have a ranking from 0 to 8, which can be used to have an overview of the resources required for addressing flood events across various areas and scales.

7 $$\begin{aligned} EI = B_{\rm it}+ P_{\rm it} \end{aligned}$$

## Data Availability

Satellite imagery from Sentinel has been downloaded from the Copernicus Agency website (https://dataspace.copernicus.eu/explore-data/data-collections/sentinel-data/). Microsoft Building Footprints free of access (https://github.com/microsoft/GlobalMLBuildingFootprints) as WPGP (https://www.worldpop.org/). Results of this work were presented at EGU 2024. This conference does not have conference proceedings, and only accepted abstracts are published.

## Code availability

Fully functional framework and a tutorial can be found at https://github.com/jeremyEudaric/SIFRAF- The flood mask was developed using Python 3.10.0. Building and population exposure analysis was performed using QGIS 3.22.
