# Correction: A satellite imagery-driven framework for rapid resource allocation in flood scenarios to enhance loss and damage fund effectiveness

**DOI:** 10.1038/s41598-026-61517-3

**Published:** 2026-07-20

**Authors:** Jeremy Eudaric, Heidi Kreibich, Andrés Camero, Kasra Rafiezadeh Shahi, Sandro Martinis, Xiao Xiang Zhu

**Affiliations:** 1 Chair of Data Science in Earth Observation, Department of Aerospace and Geodesy, Technical University of Munich, 80333 Munich, Germany; 2 Earth Observation Center, German Aerospace Center (DLR), 82234 Wessling, Germany; 3 Section Hydrology, GFZ German Research Centre for Geosciences, 14467 Potsdam, Germany; 4 Munich Center for Machine Learning, 80333 Munich, Germany

Correction to: *Scientific Reports* 10.1038/s41598-024-69977-1, published online 20 August 2024

The original version of this Article contained errors.

Equation 2 was incorrectly given as:

$$\begin{array}{*{20}c} {IoU = } & {\frac{TP}{{TP + FP + TN}}} \\ \end{array}$$

The correct Equation 2 appears below.

$$\begin{array}{*{20}c} {IoU = } & {\frac{TP}{{TP + FP + FN}}} \\ \end{array}$$

In addition, Equation 7 was incorrectly given as:

$$\begin{array}{*{20}c} {EI = B_{{{\mathrm{it}}}} + F_{{{\mathrm{it}}}} } \\ \end{array}$$

The correct Equation 7 appears below.

$$\begin{array}{*{20}c} {EI = B_{{{\mathrm{it}}}} + P_{{{\mathrm{it}}}} } \\ \end{array}$$

Finally, in the Methodology section, under the subheading ‘Flood mapping’, ‘Evaluating the inundation map’,

“This index is calculated using reflectance values from near-infrared (NIR) and shortwave infrared (SWIR) bands of Sentinel-2.”

now reads:

“This index is calculated using reflectance values from near-infrared (NIR) and Green bands of Sentinel-2.”

The original Article has been corrected.

